# Transamination-Like Reaction Catalyzed by Leucine Dehydrogenase for Efficient Co-Synthesis of α-Amino Acids and α-Keto Acids

**DOI:** 10.3390/molecules26237287

**Published:** 2021-11-30

**Authors:** Xiaoqing Mu, Xian Feng, Tao Wu, Feng Zhou, Yao Nie, Yan Xu

**Affiliations:** 1 Laboratory of Brewing Microbiology and Applied Enzymology, School of Biotechnology, Jiangnan University, Wuxi 214122, China; fx2691695431@163.com (X.F.); 7200201064@stu.jiangnan.edu.cn (T.W.); 7170201062@stu.jiangnan.edu.cn (F.Z.); ynie@jiangnan.edu.cn (Y.N.); yxu@jiangnan.edu.cn (Y.X.); 2 Key Laboratory of Industrial Biotechnology, Ministry of Education, School of Biotechnology, Jiangnan University, Wuxi 214122, China; 3 Institute of Industrial Technology, Suqian Jiangnan University, Suqian 223800, China

**Keywords:** transamination-like reaction, leucine dehydrogenase, α-amino acids, α-keto acids, co-synthesis

## Abstract

α-Amino acids and α-keto acids are versatile building blocks for the synthesis of several commercially valuable products in the food, agricultural, and pharmaceutical industries. In this study, a novel transamination-like reaction catalyzed by leucine dehydrogenase was successfully constructed for the efficient enzymatic co-synthesis of α-amino acids and α-keto acids. In this reaction mode, the α-keto acid substrate was reduced and the α-amino acid substrate was oxidized simultaneously by the enzyme, without the need for an additional coenzyme regeneration system. The thermodynamically unfavorable oxidation reaction was driven by the reduction reaction. The efficiency of the biocatalytic reaction was evaluated using 12 different substrate combinations, and a significant variation was observed in substrate conversion, which was subsequently explained by the differences in enzyme kinetics parameters. The reaction with the selected model substrates 2-oxobutanoic acid and L-leucine reached 90.3% conversion with a high total turnover number of 9.0 × 10^6^ under the optimal reaction conditions. Furthermore, complete conversion was achieved by adjusting the ratio of addition of the two substrates. The constructed reaction mode can be applied to other amino acid dehydrogenases in future studies to synthesize a wider range of valuable products.

## 1. Introduction

*α-*Amino acids, especially natural *α*-amino acids, are the main building blocks of proteins and precursors for the synthesis of secondary metabolites, and play an indispensable role in the progress of various areas of life sciences [1]. *α-*keto acids, which possess a keto group adjacent to the carboxylic acid, also play a central role in cell metabolism [2]. Moreover, both *α*-amino acids and *α*-keto acids are important intermediates in the pharmaceutical, food, cosmetic, agricultural, and animal feed industries [3,4,5,6]. The synthesis of *α*-amino acids and *α*-keto acids can be achieved using chemical [7,8,9,10] or biocatalytic methods [11,12,13,14]. Compared with chemical methods, biocatalytic synthesis is an attractive approach providing the advantages of low cost, mild conversion conditions, and green production credentials, and has gained increasing research interest [15,16,17,18].

*α-*Amino acids and *α*-keto acids can be starting substrates for each other’s synthesis through transamination reactions [19,20]. The transaminase enzyme mediates the transfer of an amino group from the amino donor to the acceptor for the simultaneous synthesis of an *α*-amino acid and *α*-keto acid in a single reaction with a high atom and step economy, making it the biocatalyst of choice for academic research as well as industrial applications [21]. However, the substrate specificities and catalytic efficiency of transaminase in reversible reactions need to be investigated thoroughly to meet the demands of industrial processes [22]. Amino acid dehydrogenase is another potential enzyme that can be used for α-amino acid or α-keto acid synthesis via reductive amination or oxidative deamination reaction [23,24,25,26,27]. However, amino acid dehydrogenase thermodynamically favors the reductive amination reaction of α-keto acids, which uses the expensive coenzyme NADH as a reducing agent [28,29]. This necessitates the coupling of the amino acid dehydrogenase with an additional enzyme such as glucose dehydrogenase (GDH) or formate dehydrogenase (FDH) to regenerate NADH and improve the total turnover number (TTN) [30,31,32,33], which increases the cost and complexity of the process.

**Figure 1:**
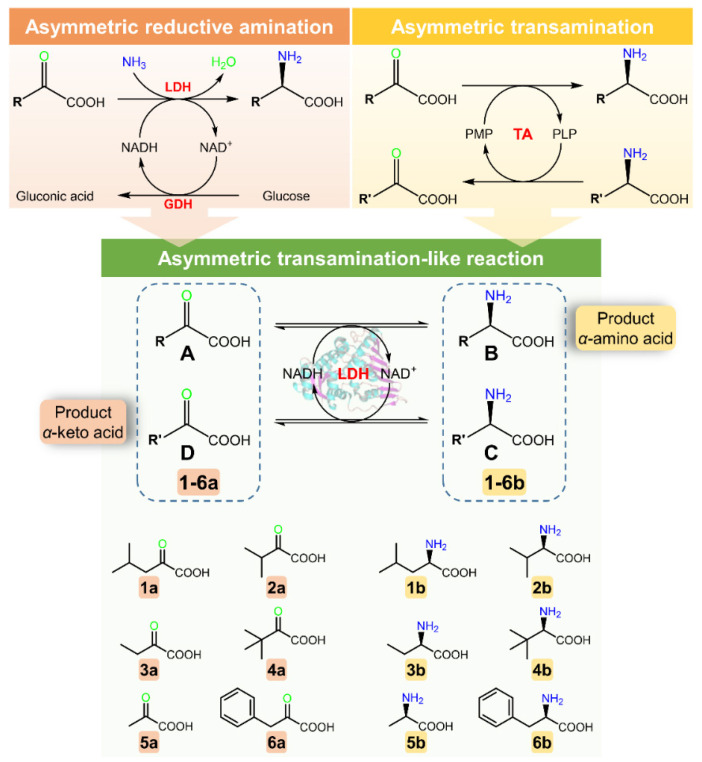
Schematic representation of reaction pathway of transamination-like reaction.

Thus, to facilitate the simultaneous synthesis of *α*-amino acids and *α*-keto acids in a single self-sustaining reaction, we attempted to construct a novel transamination-like reaction catalyzed by an amino acid dehydrogenase by coupling the oxidative deamination of an amino acid and the reductive amination of a keto acid (Figure 1). In this reaction, the rate of the thermodynamically unfavorable oxidation reaction matches that of the reduction reaction driven by the coenzyme NADH. Moreover, no coenzyme regeneration system is needed in this reaction owing to the complementary coenzyme dependence of the reduction and oxidation reactions. Both the reduced and oxidized forms of the coenzyme are quickly utilized in the transamination-like reaction until the reaction equilibrium is reached.

Leucine dehydrogenase (LDH) uses NADH or NAD^+^ to catalyze the reversible transformation between the natural substrates 4-methyl-2-oxopentanoic acid and L-leucine [24,26], and has been reported to be a crucial enzyme in the synthesis of commercially valuable *α*-amino acids such as L-2-aminobutyric acid [28,34] and L-tert-leucine [30,31]. In the present study, LDH from *Bacillus cereus* [29] was chosen to construct a transamination-like reaction for the simultaneous synthesis of *α*-amino acids and *α*-keto acids (Figure 1). Twelve combinations of substrates, selected based on LDH specificity, were evaluated for transformation via the single-step transamination-like reaction. Enzyme kinetics were analyzed to clarify the effect of the different combinations of amino acceptors and donors on the eventual conversion. Furthermore, the reaction conditions and substrate proportions were optimized for maximum conversion of the model substrates 2-oxobutanoic acid (3a) and L-leucine (1b).

## 2. Results and Discussion

### 2.1. Substrate Specificity of LDH toward α-Amino Acids and α-Keto Acids

**Figure 2:**
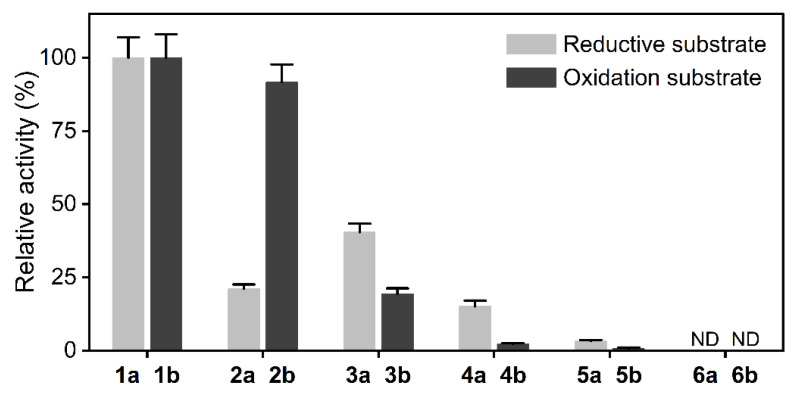
Relative activity of leucine dehydrogenase on α-amino acids and α-keto acids.

To characterize the substrate specificity of LDH, α-keto acids (4-methyl-2-oxopentanoic acid, 1a; 3-methyl-2-oxobutanoic acid, 2a; 2-oxobutanoic acid, 3a; 3,3-dimethyl-2-oxobutanoic acid, 4a; 2-oxopropanoic acid, 5a; 2-oxo-3-phenylpropanoic acid, 6a) and α-amino acids (L-leucine, 1b; L-valine, 2b; L-2-aminobutyric acid, 3b; L-*tert*-leucine, 4b; L-alanine, 5b; L-phenylalanine, 6b) were chosen for catalytic activity investigation. Consistent with previous literature reports, LDH showed measurable activity toward the aliphatic α-amino acids 1a to 5a and no activity toward the aromatic 2-oxo-3-phenylpropanoic acid (6a) (Figure 2) in the reduction direction [35]. Moreover, LDH reversibly catalyzed the conversion of a series of aliphatic α-keto acids (1b to 5b) to α-amino acids, (Figure 2) as an aminotransferase. However, α-aminotransferases usually require amino acids as amino donors for α-keto acid reduction, and the conversion rate of the amino transfer reaction is restricted by thermodynamic equilibrium. In contrast, LDH can use the cheap and easily available ammonia as an amino donor to catalyze the reductive amination of α-keto acids with a high atom economy.

Activity was measured with 5 mM substrate and 0.2 mM coenzyme at 30 °C and carried out at a 200-μL scale in 96-well microtiter plates by monitoring the initial decrease or increase velocity of the absorbance at 340 nm. The specific activity of the reductive substrates and the oxidation substrates were calculated with 1a and 1b as the maximum activity, respectively. ND: no detectable activity. All determinations were performed in triplicate, and error bounds represent ± sd.

### 2.2. Conversion of Single-Step Oxidation and Reduction Reactions Catalyzed by LDH

**Figure 3:**
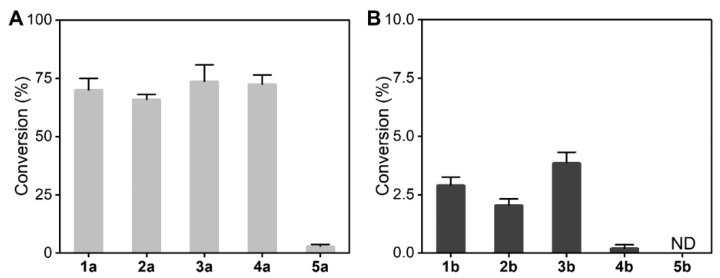
Single-step conversion experiment. (**A**): Conversion of different α-keto acids by LDH, (**B**): conversion of corresponding α-amino acids by LDH.

The extent of conversion during the reaction was not only decided by the specific activity of the LDH enzyme, but also influenced by the reaction conditions. Both the reduction reactions of 5 α-keto acids and oxidation reactions of the five corresponding α-amino acids were investigated. As shown in Figure 3, the conversion of the single-step oxidation reaction catalyzed by LDH were low, and did not exceed approximately 3%, whereas the conversion of the single-step reduction reaction reached approximately 70%. The conversion of the reduction reaction was nearly 23-fold higher than that of oxidative deamination, which indicated that LDH favors the reductive amination of α-keto acid to the corresponding α-amino acid.

Reductive amination conditions: The total volume of the biocatalytic reaction mixture was 2 mL. It contained 10 mM α-keto acids, 10 mM NAD^+^, 1.0 mg·mL^−1^ of purified LDH, and 1 M NH_4_Cl/NH_4_OH buffer (pH 9.5). Oxidative deamination conditions: The total volume of the biocatalytic reaction mixture was 2 mL. It contained 10 mM α-amino acids, 10 mM NADH, 1.0 mg·mL^−1^ of purified LDH, and 0.1 M Tris-HCl buffer (pH 8.5). The reaction mixture was incubated at 30 °C with shaking at 200 rpm for 12 h. All determinations were performed in triplicate, and error bounds represent ± sd.

### 2.3. Effect of Different Combinations of Amino Group Donor and Acceptor on Conversion Rate

Although LDH showed low activities and conversion on α-amino acids, the enzyme could be used to oxidize the amino acids to produce keto acids when driven by coupling a high-efficiency coenzyme regeneration system. Based on the mechanism of the transamination reaction, a substrate-coupled transamination-like reaction catalyzed by LDH was established using the reduction of keto acid as a coupled coenzyme regeneration system to promote the oxidation reaction. The coenzyme dependence of reductive amination was complementary to that of oxidative deamination, providing an easy route for sustained coenzyme regeneration.

**Figure 4:**
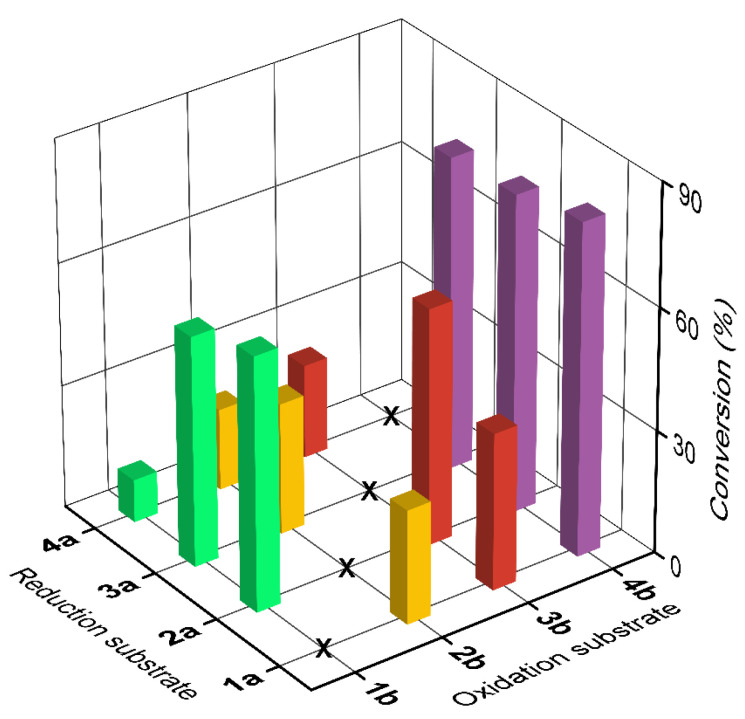
Conversion for reactions employing combinations of oxidation substrate and reduction substrate.

Based on the substrate specificity of LDH and conversion of single-step oxidation or reduction reactions, keto acid substrates (1a, 2a, 3a, and 4a) and the corresponding amino acid substrates (1b, 2b, 3b, and 4b) were chosen to design 12 combinations of amino group donors and acceptors. The conversion of reactions employing the different substrate combinations are shown in Figure 4. On coupling with the reductive amination reaction, the conversion of most of the oxidation reactions increased to varying degrees compared with those of the single-step reaction. These results confirmed the feasibility of using the LDH-catalyzed transamination-like reaction for the simultaneous synthesis of α-amino acids and α-keto acids.

When 4b participated in the reaction, the conversion of the all keto acid substrates tested (1a, 2a, and 3a) improved considerably. The conversion of 4b reached 77.8%, which is 77 times higher than its conversion in the single-step oxidation reaction. In contrast, when 4a participated in the reaction, the conversion of the different amino acid substrates (1b, 2b, and 3b) did not show a substantial increase. Nevertheless, the conversion of 4b reached 22.6%, which is still 7.8 times higher than that achieved in the single-step oxidation reaction.

The total volume of the biocatalytic reaction mixture was 2 mL. It contained 10 mM α-amino acids, 10 mM α-keto acids, 0.2 mM NAD^+^, 1.0 mg·mL^−1^ of purified LDH, 1 M NH_4_Cl, and 0.1 M Tris-HCl buffer (pH 9.0). The reaction mixture was incubated at 30 °C with shaking at 200 rpm for 12 h. All determinations were performed in triplicate, and error bounds represent ± sd.

### 2.4. Kinetic Parameters of LDH on Different Substrate

To explore the reasons for the difference in conversion of different substrate combinations, the kinetic parameters of LDH for the reduction of 1a, 2a, 3a, and 4a and oxidation of 1b, 2b, 3b, and 4b were investigated.

As shown in Table 1, the *k*_cat_/*K*_m_ of LDH for different keto acids decreased in the sequence 1a > 2a >3a > 4a in the reductive amination reaction, and *k*_cat_/*K*_m_ decreased in a similar sequence for the corresponding amino acids in the oxidative deamination reaction. The *k*_cat_/*K*_m_ values of the reduction reactions were 2–3 orders of magnitude higher than that of the oxidation reactions, which explains why LDH favors catalyzing the reductive amination reaction. The kinetic parameters of different reduction or oxidation substrates and the conversion of reactions employing different substrate combinations suggested that the transamination-like reaction followed the following rules.

As shown in Figure 1, the conversion efficiency of the transamination-like reaction was determined by the ratio of the *k*_cat_/*K*_m_ of the reduction reaction substrate (A) and the *k*_cat_/*K*_m_ of the reduction product corresponding to the oxidation reaction substrate (D). A higher ratio resulted in a higher conversion efficiency. If the catalytic efficiency in the reaction from A to B was greater than that in the reaction from D to C, substrate A preferentially bound the NADH coenzyme to prevent the reduction reaction of product D.

### 2.5. Optimization of Conditions for the Transamination-Like Reaction

Through comprehensive consideration of conversion and product value, the substrate combination of 2-oxobutyric acid (3a) and L-leucine (1b) was investigated as a substrate mode reaction. The reaction conditions, including coenzyme concentration, NH_4_^+^ concentration, pH, and substrate concentration, were optimized.

**Figure 5:**
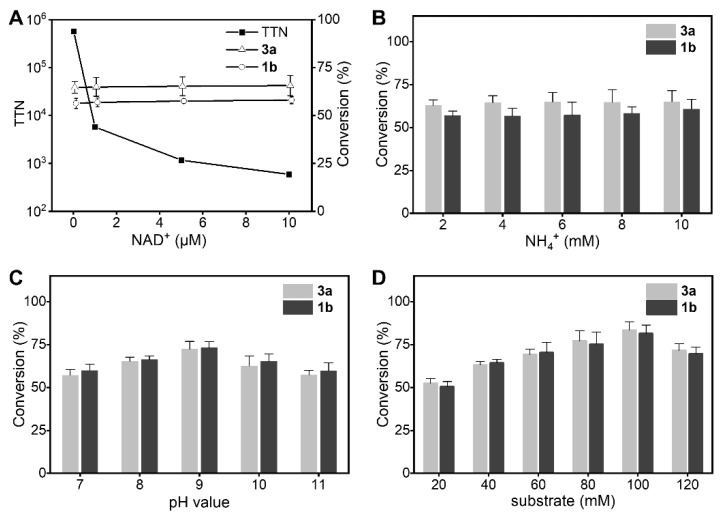
Effect of NAD^+^ concentration (**A**), NH_4_^+^ concentration (**B**), pH value (**C**), and substrate concentration (**D**) in the transamination-like reaction involving substrates 3a and 1b.

The effect of NAD^+^ concentration on the conversion of the transamination-like reaction system is shown in Figure 5A. When the coenzyme concentration varied from 0.01 µM to 10 µM, the TTN of the reaction dropped sharply, whereas the conversion of 3a and 1b did not change substantially. When the concentration of coenzyme was 0.01 µM, the conversion of 3a and 1b reached 58.8% and 65.6%, respectively, with a high TTN of 5.9 × 10^5^. This shows that the concentration of coenzyme has a negligible impact on the final conversion in transamination-like reactions, as both the oxidized and reduced forms of the coenzyme would be consumed quickly by oxidation or reduction reactions. Thus, a coenzyme self-cycle was built in transamination-like reactions.

Studying the effect of NH_4_^+^ concentrations from 2 mM to 10 mM revealed that NH_4_^+^ had almost no effect on the transamination-like reaction (Figure 5B). NH_4_^+^ is the product of the oxidation reaction and can be used as an amino group donor during the transamination-like reaction. Therefore, 2 mM was decided as the optimal NH4^+^ concentration for the transamination reaction.

The reaction pH was varied from 7.0 to 11.0 to evaluate its effect on the conversion. As shown in Figure 5C, the conversion rate increased with an increase in the pH range from 7.0 to 9.0. Maximum conversions of 73.2% and 72.3% were achieved with 3a and 1b, respectively, at pH 9.0. The conversion rate dropped beyond pH 9.0, which may be due to enzyme denaturation and inactivation at high pH values. Therefore, pH 9.0 was selected as the optimal pH value for the transamination reaction.

As shown in Figure 5D, the effect of initial substrate concentration on conversion was studied in the range 20–100 mM. The conversion of 3a and 1b reached maximum values of 83.4% and 81.5%, respectively, with 100 mM initial substrate concentration. The conversion decreased when a substrate concentration of 120 mM was used, indicating that high substrate concentrations inhibit the reaction. Therefore, 100 mM was chosen as the optimal substrate concentration for further transamination-like reactions.

The total volume of the biocatalytic reaction mixture was 2 mL. It contained purified LDH (1.0 mg·mL^−1^), NAD^+^ (0.01–10 μM), NH_4_Cl (2–10 mM), tris-HCl buffer (pH 7.0–9.0) or NH_4_Cl/NH_4_OH (pH 10.0–11.0), and substrates (20–120 mM). The reaction mixture was incubated at 30 °C with shaking at 200 rpm for 12 h. All determinations were performed in triplicate, and error bounds represent ± sd.

**Figure 6:**
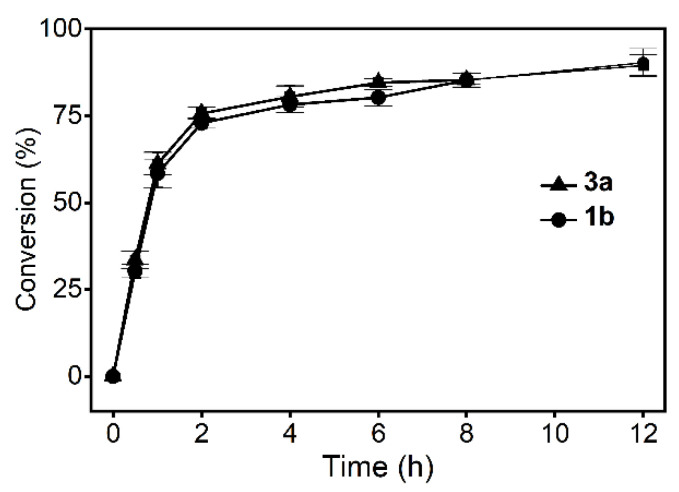
Reaction progress curve under optimal conditions.

Under the optimal reaction conditions, the initial reaction rate was quite high and the conversion reached 75% in the first 2 h. Subsequently, the reaction slowed down, with the final conversion of 1b and 3a reaching 90.3% and 89.5%, respectively. In addition, the TTN increased from 5.9 × 10^5^ to 9.0 × 10^6^ compared with the previous reaction. However, the conversion did not reach 100% even on extending the reaction time, probably owing to the influence of thermodynamic equilibrium (Figure 6).

The total volume of the biocatalytic reaction mixture was 2 mL. It contained purified LDH (1.0 mg·mL^−1^), NAD^+^ (0.01 μM), NH_4_Cl (2 mM), tris-HCl buffer (pH 9.0), and substrates (100 mM). The reaction mixture was incubated at 30 °C with shaking at 200 rpm for 0–12 h. All determinations were performed in triplicate, and error bounds represent ± sd.

### 2.6. Shifting Reaction Equilibrium by Adjusting the Substrate Ratio

**Figure 7:**
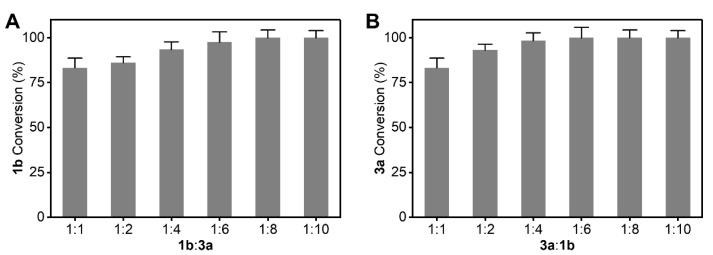
Effect of different substrate addition ratio toward the conversion rate. Effect of different substrate addition ratio toward the conversion rate of 1b (**A**) and 3a (**B**).

Even after optimization of the reaction conditions, complete conversion was still not achieved. Adjusting the substrate ratio is a common strategy to improve the reaction conversion in double substrate reactions such as transamination reactions. Therefore, we tested different ratios of the two substrates in an effort to shift the reaction equilibrium and further enhance the conversion. As shown in Figure 7, the conversion rates of 1b and 3a reached 100% when the molar ratios of 1b to 3a were 1:8 and 6:1, respectively. These results indicated that adjusting the substrate ratio was also effective in improving conversion in transamination-like reactions.

The total volume of the biocatalytic reaction mixture was 2 mL. It contained purified LDH (1.0 mg·mL^−1^), NAD^+^ (0.01 μM), NH_4_Cl (2 mM), tris-HCl buffer (pH 9.0), and two substrates which were added in different ratios. The reaction mixture was incubated at 30 °C with shaking at 200 rpm for 0–12 h. All determinations were performed in triplicate, and error bounds represent ± sd.

## 3. Materials and Methods

### 3.1. Strains, Plasmids, and Chemicals

Leucine dehydrogenase (LDH) from *Bacillus cereus* was in our laboratory. Plasmid pET-28a (+) and *Escherichia coli* BL21 (DE3) were procured from Novagen (Nanjing, China) and used as the gene expression vector and host, respectively. Kanamycin, isopropyl-*β*-D-thiogalactopyranoside (IPTG), 1-fluoro-2,4-dinitrophenyl-5-L-alanineamide (FDAA), NAD^+^, and NADH were obtained from TCI (Shanghai, China). α-keto acids (4-methyl-2-oxopentanoic acid, 3-methyl-2-oxobutanoic acid, 2-oxobutanoic acid, 3,3-dimethyl-2-oxobutanoic acid, 2-oxopropanoic acid, 2-oxo-3-phenylpropanoic acid) and α-amino acids (L-leucine, L-valine, L-2-aminobutyric acid, L-*tert*-leucine, L-alanine, L-phenylalanine) were purchased from J&K Chemical (Beijing, China). All other chemicals were purchased from commercial sources, at least reagent grade, and were used without further purification.

### 3.2. Protein Expression and Purification

*Escherichia coli* BL21 (DE3) cells containing recombinant pET-28a (+) plasmids were cultured in Luria-Bertani (LB) medium with kanamycin (50 mg·L^−1^) at 37 °C and with shaking at 200 rpm for 2 h. Enzyme expression was induced with IPTG (final concentration = 0.2 mM) when the bacteria reached OD_600_ = 0.6–0.8. The cells were incubated at 20 °C and with shaking at 200 rpm for 12 h. *His*-tagged enzymes were purified with an ÄKTA purifier system (GE Healthcare, Little Chalfont, UK). The cells were resuspended in buffer A (100 mM potassium phosphate, 150 mM NaCl, and 20 mM imidazole; pH 7.5) and disrupted with an ultrasonic cell disruption system (SCIENTZ-IID; Ningbo Scientz Biotechnology, Ningbo, Zhejiang, China) followed by centrifugation at 12,000 rpm and 4 °C for 30 min to remove cell debris and obtain cell-free extracts. The latter were passed through a 0.22-μm filter and loaded into a standard Ni-NTA affinity column (Thermo Fisher Scientific, Waltham, MA, USA) pre-equilibrated with buffer A. The column was then gradient-eluted with buffer B (100 mM potassium phosphate, 150 mM NaCl, and 500 mM imidazole; pH 7.5). Eluted fraction purity was determined by SDS-PAGE.

### 3.3. Enzyme Activity Assay

Enzyme activity was measured at 340 nm and at 30 °C using a MultiSkan GO UV-spectrometer (Thermo Fisher Scientific). The standard assay mixture for the oxidative deamination reaction contained 5 mM α-amino acids, 0.1 M Tris-HCl buffer (pH 8.5), and 0.2 mM NAD^+^. The reductive amination reaction was carried out in 1 M NH_4_Cl/NH_4_OH buffer (pH 9.5) with 5 mM α-keto acids and 0.2 mM NADH. Both reactions were performed in a final volume of 200 μL and initiated by the addition of limiting amounts of enzyme. One unit of enzyme activity (1 U) was defined as the amount of enzyme which catalyzes the production (or consumption) of 1 μmol of NADH per min under the standard assay conditions. Three sets of parallel experiments were performed for each experimental condition. All determinations were performed in triplicate, and error bounds represent ± sd.

### 3.4. Kinetic Parameters Analysis

According to the enzymatic reaction equation, Michaelis-Menten equation: V = (*V*_max_ ∗ S)/(*K*_m_ + S), when V = 0.5 *V*_max_, *K*_m_ = [S]. It can be seen that *K*_m_ is equal to the substrate concentration when the initial rate of enzymatic reaction is half of the maximum rate *V*_max_. *V*_max_ represents the maximum reaction rate at a certain amount of enzyme. Kinetic parameters with respect to substrate α-keto acids were evaluated in NH_4_Cl/NH_4_OH buffer (1 M; pH 9.5) at 30 °C in the presence of 1–100 mM substrates and 0.5 mM NADH with the purified enzymes. Kinetic parameters with respect to substrate α-amino acids were evaluated in Tris-HCl buffer (0.1 M; pH 8.5) at 30 °C in the presence of 1–100 mM substrates and 0.5 mM NAD^+^ with the purified enzymes. Kinetic parameters with respect to NADH were evaluated in NH_4_Cl/NH_4_OH buffer (1 M; pH 9.5) at 30 °C in the presence of 0.1–0.5 mM NADH and 20 mM 4-methyl-2-oxopentanoic acid with the purified enzymes. Kinetic parameters with respect to NAD^+^ were evaluated in Tris-HCl buffer (0.1 M; pH 8.5) at 30 °C in the presence of 0.1–0.5 mM NAD^+^ and 20 mM L-leucine with the purified enzymes. All reactions were performed in a final volume of 200 μL. After incubation at 30 °C for 2 min, the amount of enzyme solution was added to measure the absorbance change at a wavelength of 340 nm, and the enzyme activity of LDH was calculated. Three sets of parallel experiments were performed for each experimental condition. All determinations were performed in triplicate, and error bounds represent ± sd.

### 3.5. Reaction Conditions Optimization

The total volume of the biocatalytic reaction mixture was 2 mL. It contained purified LDH (1.0 mg·mL^−1^), NAD^+^ (0.01–10 μM), NH_4_Cl (2–10 mM), tris-HCl buffer (pH 7.0–9.0) or NH_4_Cl/NH_4_OH (pH 10.0–11.0), and substrates (20–120 mM). The reaction mixture was incubated at 30 °C with shaking at 200 rpm for 12 h. Samples (200 μL) were drawn at intervals and alkalized with 200 μL NaOH (10 M) to terminate the reaction. The solutions were then subjected to High Performance Liquid Chromatography (HPLC). All determinations were performed in triplicate, and error bounds represent ± sd.

### 3.6. Reaction Progress Monitoring

The total volume of the biocatalytic reaction mixture was 2 mL. It contained purified LDH (1.0 mg·mL^−1^), NAD^+^ (0.01 μM), NH_4_Cl (2 mM), tris-HCl buffer (pH 9.0), and substrates (100 mM). The reaction mixture was incubated at 30 °C with shaking at 200 rpm for 0–12 h. Samples (200 μL) were drawn at intervals and alkalized with 200 μL NaOH (10 M) to terminate the reaction. The solutions were then subjected to High Performance Liquid Chromatography (HPLC). All determinations were performed in triplicate, and error bounds represent ± sd.

### 3.7. HPLC Analysis

The substrates conversion was determined with an Agilent 1260 HPLC (Agilent Technologies, Santa Clara, CA, USA). 1-fluoro-2,4-dinitrophenyl-5-L-alanine amide (FDAA) was applied to distinguish the absolute configuration of α-amino acid products through pre-column derivatizing reaction [36,37]. The conversion rate of α-amino acids was determined after derivatization with FDAA. A 5-μL reaction sample, 4 μL of 1 M NaHCO_3_, and 20 μL of 1% (*w*/*v*) FDAA in acetone were mixed and heated at 40 °C for 60 min. Then, 4 μL of 1 M HCl and 467 μL of 40% (*v*/*v*) aqueous acetonitrile were added to the mixture. The latter was then passed through a 0.22-μm filter and subjected to HPLC. The conditions for determining the conversion rate of α-amino acids were as follows: Develosil^®^ ODS-UG-5 column (5 μm; 150 mm × 4.6 mm) (Phenomenex); mobile phase B (5% (*v*/*v*) acetonitrile, 0.05% (*v*/*v*) trifluoroacetic acid, and 1% (*v*/*v*) methanol); mobile phase C (60% (*v*/*v*) acetonitrile, 0.05% (*v*/*v*) trifluoroacetic acid, and 1% (*v*/*v*) methanol); linear gradient from 0%–100%; mobile phase C, 45 min at 30 °C; flow rate, 1.0 mL·min^−1^; injection volume, 20 μL; and UV detection at 340 nm. The conditions for determining the α-keto acids conversion rate were as follows: Diamonsil C18(2) column (5 μm; 250 mm × 4.6 mm) (Phenomenex, Torrance, CA, USA); mobile phase A (55% (*v*/*v*) methanol plus 0.1% (*v*/*v*) trifluoroacetic acid), 20 min at 30 °C; flow rate, 0.8 mL·min^−1^; injection volume, 10 μL; and UV detection at 230 nm. All determinations were performed in triplicate, and error bounds represent ± sd.

## 4. Conclusions

In this study, a novel transamination-like reaction was constructed based on the characteristic catalytic property of LDH to enable the simultaneous synthesis of target α-amino acids and α-keto acids without the need for a separate coenzyme regeneration system involving GDH or FDH. The thermodynamically unfavorable oxidation reaction was driven by the reduction reaction with the consumption of NADH. As a result, a 77-fold higher conversion was achieved from the transamination-like reaction compared with that achieved with the single-step oxidation reaction. Based on the analysis of kinetic parameters, we were able to explain the effect of combinations of amino group donors and acceptors on substrate conversion. Finally, the conditions of the transamination-like reaction were investigated using 3a and 1b as model substrates. Under optimal conditions (pH 9.0, 100 mM substrate, 0.1 µM NAD^+^, and 2 mM NH_4_^+^), the conversion of 1b increased from 42.0% to 90.3% and the TTN increased from 5.9 × 10^5^ to 9.0 × 10^6^. Furthermore, complete conversion to the target product was achieved by adjusting the substrate ratio. This artificial design of transamination-like reactions for the simultaneous production of α-amino acids and α-ketone acids can be applied to other types of amino acid dehydrogenases to expand the substrate scope and synthesize a wider range of valuable products.

## Figures and Tables

**Table 1:** Kinetic parameters of His-tagged LDH for both oxidative deamination and reductive amination.

Substrate	*K*_m_ (mM)	*k*_cat_ (s^−1^)	*k*_cat_/*K*_m_ (mM·s^−1^)
**1a**	1.16 ± 0.37	167.25 ± 10.23	143.69 ± 6.36
**2a**	1.41 ± 0.19	73.20 ± 4.17	51.92 ± 1.57
**3a**	2.38 ± 0.54	62.29 ± 4.32	26.14 ± 0.58
**4a**	9.15 ± 1.23	16.79 ± 2.56	1.84 ± 0.32
**1b**	0.86 ± 0.21	0.25 ± 0.03	0.29 ± 0.03
**2b**	2.31 ± 0.11	0.17 ± 0.01	0.07 ± 0.001
**3b**	30.04 ± 1.39	0.16 ± 0.06	0.005 ± 0.001
**4b**	16.26 ± 0.86	0.05 ± 0.01	0.003 ± 0.002
NAD^+^	0.25 ± 0.04	1.07 ± 0.249	2.08 ± 0.16
NADH	0.19 ± 0.03	2.90 ± 0.78	5.64 ± 0.23

Steady-state kinetic parameters were determined by varying the concentration of the substrate (0.1–50 mM) to be measured in the presence of fixed concentrations of the coenzyme (0.5 mM NAD^+^ in the oxidative deamination and 0.5 mM NADH) and cosubstrate (1 M ammonia in the reductive amination) at 30 °C and carried out at a 200-μL scale in 96-well microtiter plates by monitoring the initial decrease or increase velocity of the absorbance at 340 nm. All determinations were performed in triplicate, and error bounds represent ± sd.

## Data Availability

All datasets presented in this study are included in the article.

## References

[1] Najera C., Sansano J.M. (2007). Catalytic asymmetric synthesis of alpha-amino acids. Chem. Rev..

[2] Basak S., Nader S., Mansy S.S. (2021). Protometabolic reduction of NAD(+) with alpha-keto acids. JACS Au..

[3] Song W., Xu X., Gao C., Zhang Y., Wu J., Liu J., Chen X., Luo Q., Liu L. (2020). Open gate of corynebacterium glutamicum threonine deaminase for efficient synthesis of bulky alpha-keto acids. ACS Catal..

[4] Galkin A., Kulakova L., Yoshimura T., Soda K., Esaki N. (1997). Synthesis of optically active amino acids from α-keto acids with *Escherichia coli* cell expressing heterologous genes. Appl. Environ. Microbiol..

[5] Penteado F., Lopes E.F., Alves D., Perin G., Jacob R.G., Lenardao E.J. (2019). Alpha-keto acids: Acylating agents in organic synthesis. Chem. Rev..

[6] Chun S.W., Narayan A.R.H. (2020). Biocatalytic, stereoselective deuteration of alpha-amino acids and methyl esters. ACS Catal..

[7] Moriwaki H., Resch D., Li H., Ojima I., Takeda R., Luis Acena J., Soloshonok V. (2014). Inexpensive chemical method for preparation of enantiomerically pure phenylalanine. Amin. Acids..

[8] Ogo S., Uehara K., Abura T., Fukuzumi S. (2004). pH-dependent chemoselective synthesis of alpha-amino acids. Reductive amination of alpha-keto acids with ammonia catalyzed by acid-stable iridium hydride complexes in water. J. Am. Chem. Soc..

[9] Kang H.Y., Ji Y.M., Yu Y.K., Yu J.Y., Lee Y., Leet S.J. (2003). Synthesis of alpha-ketobutyrolactones and gamma-hydroxy-alpha-keto acids. Bull. Korean Chem Soc..

[10] Xie Y., Lou R., Zhi L., Mi A., Jiang Y. (2000). DPAMPP in catalytic asymmetric reactions: Enantioselective synthesis of L-homophenylalanine. Tetrahedron Asymmetry.

[11] Xue Y.-P., Cao C.-H., Zheng Y.-G. (2018). Enzymatic asymmetric synthesis of chiral amino acids. Chem. Soc. Rev..

[12] Simon R.C., Mutti F.G., Kroutil W. (2013). Biocatalytic synthesis of enantiopure building blocks for pharmaceutical. Drug Discov. Today..

[13] Turner N.J., O’Reilly E. (2013). Biocatalytic retrosynthesis. Nat. Chem. Biol..

[14] Bornscheuer U.T., Huisman G.W., Kazlauskas R.J., Lutz S., Moore J.C., Robins K. (2012). Engineering the third wave of biocatalysis. Nature.

[15] Bornscheuer U.T. (2016). Biocatalysis: Successfully crossing boundaries. Angew. Chem. Int. Ed..

[16] Yi D., Bayer T., Badenhorst C.P.S., Wu S., Doerr M., Höhne M., Bornscheuer U.T. (2021). Recent trends in biocatalysis. Chem. Soc. Rev..

[17] Sheldon R.A., Woodley J.M. (2018). Role of biocatalysis in sustainable chemistry. Chem. Rev..

[18] Savile C.K., Janey J.M., Mundorff E.C., Moore J.C., Tarn S., Jarvis W.R., Colbeck J.C., Krebber A., Fleitz F.J., Brands J. (2010). Biocatalytic asymmetric synthesis of chiral amines from ketones applied to sitagliptin manufacture. Science.

[19] Slabu I., Galman J.L., Lloyd R.C., Turner N.J. (2017). Discovery, engineering, and synthetic application of transaminase biocatalysts. ACS Catal..

[20] Truppo M.D., Rozzell J.D., Moore J.C., Turner N.J. (2009). Rapid screening and scale-up of transaminase catalysed reactions. Org. Biomol. Chem..

[21] Truppo M.D., Strotman H., Hughes G. (2012). Development of an immobilized transaminase capable of operating in organic solvent. ChemCatChem.

[22] Guo F., Berglund P. (2017). Transaminase biocatalysis: Optimization and application. Green Chem..

[23] Koike K., Hakamada Y., Yoshimatsu T., Kobayashi T., Ito S. (1996). NADP-specific glutamate dehydrogenase from *Alkaliphilic Bacillus sp.* KSM-635: Purification and enzymatic properties. Biosci. Biotechnol. Biochem..

[24] Li H.M., Zhu D.M., Hyatt B.A., Malik F.M., Biehl E.R., Ling H. (2009). Cloning, protein sequence clarification, and substrates specificity of a leucine dehydrogenase from *Bacillus sphaericus* ATCC4525. Appl. Biochem. Biotechnol..

[25] Asano Y., Yamada A., Kato Y., Yamaguchi K., Hibino Y., Hirai K., Kondo K. (1990). Enantioselective synthesis of (*S*)-amino acids by phenylalanine dehydrogenase from *Bacillus sphaericus*: Use of aatural and recombinant enzymes. J. Org. Chem..

[26] Ouyang S., Li X., Sun X., Ouyang J., Yong Q. (2020). A thermostable leucine dehydrogenase from *Bacillus coagulans* NL01: Expression, purification and characterization. Process. Biochem..

[27] Yousefi F., Ataei F., Arab S.S., Hosseinkhani S. (2017). Increase of *Bacillus badius* phenylalanine dehydrogenase specificity towards phenylalanine substrate by site-directed mutagenesis. Arch. Biochem Biophys..

[28] Xu J.-M., Cheng F., Fu F.-T., Hu H.-F., Zheng Y.-G. (2017). Semi-rational engineering of leucine dehydrogenase for L-2-aminobutyric acid production. Appl. Biochem. Biotechnol..

[29] Zhou J.P., Wang Y.L., Chen J.J., Xu M.J., Yang T.W., Zheng J.X., Zhang X., Rao Z.M. (2019). Rational engineering of *Bacillus cereus* leucine dehydrogenase towards α-keto Acid reduction for improving unnatural amino acid production. Biotechnol. J..

[30] Zhou F., Mu X., Nie Y., Xu Y. (2021). Enhanced catalytic efficiency and coenzyme affinity of leucine dehydrogenase by comprehensive screening strategy for L-*tert*-leucine synthesis. Appl. Microbiol. Biotechnol..

[31] Luo W., Zhu J., Zhao Y., Zhang H., Yang X., Liu Y., Rao Z., Yu X. (2020). Cloning and expression of a novel leucine dehydrogenase: Characterization and L-tert-leucine production. Front. Bioeng. Biotechnol..

[32] Yin X.J., Liu Y.Y., Meng L.J., Zhou H.S., Wu J.P., Yang L.R. (2019). Rational molecular engineering of glutamate dehydrogenases for enhancing asymmetric reductive amination of bulky *α*-keto acids. Adv. Synth. Catal..

[33] Wu T., Mu X., Xue Y., Xu Y., Nie Y. (2021). Structure-guided steric hindrance engineering of *Bacillus badius* phenylalanine dehydrogenase for efficient L-homophenylalanine synthesis. Biotechnol. Biofuels..

[34] Chen J., Zhu R., Zhou J., Yang T., Zhang X., Xu M., Rao Z. (2021). Efficient single whole-cell biotransformation for L-2-aminobutyric acid production through engineering of leucine dehydrogenase combined with expression regulation. Bioresour Technol..

[35] Jin J.-Z., Chang D.-L., Zhang J. (2011). Discovery and application of new bacterial strains for asymmetric synthesis of L-tert-butyl leucine in high enantioselectivity. Appl. Biochem. Biotechnol..

[36] Krishnamurthy T. (1994). Chirality in microcystins. J. Am. Soc. Mass Spectrom..

[37] B’Hymer C., Montes-Bayon M., Caruso J.A. (2003). Marfey’s reagent: Past, present, and future uses of 1-fluoro-2,4-dinitrophenyl-5-L-alanine amide. J. Sep. Sci..

